# Mechanical Properties of the Compass Depressors of the Sea-Urchin *Paracentrotus lividus* (Echinodermata, Echinoidea) and the Effects of Enzymes, Neurotransmitters and Synthetic Tensilin-Like Protein

**DOI:** 10.1371/journal.pone.0120339

**Published:** 2015-03-18

**Authors:** Iain C. Wilkie, Dario Fassini, Emanuele Cullorà, Alice Barbaglio, Serena Tricarico, Michela Sugni, Luca Del Giacco, M. Daniela Candia Carnevali

**Affiliations:** 1 Department of Life Sciences, Glasgow Caledonian University, Glasgow, Scotland, United Kingdom; 2 Dipartimento di Bioscienze, Università degli Studi di Milano, Milano, Italy; Stazione Zoologica Anton Dohrn, Naples, ITALY

## Abstract

The compass depressors (CDs) of the sea-urchin lantern are ligaments consisting mainly of discontinuous collagen fibrils associated with a small population of myocytes. They are mutable collagenous structures, which can change their mechanical properties rapidly and reversibly under nervous control. The aims of this investigation were to characterise the baseline (i.e. unmanipulated) static mechanical properties of the CDs of *Paracentrotus lividus* by means of creep tests and incremental force-extension tests, and to determine the effects on their mechanical behaviour of a range of agents. Under constant load the CDs exhibited a three-phase creep curve, the mean coefficient of viscosity being 561±365 MPa.s. The stress-strain curve showed toe, linear and yield regions; the mean strain at the toe-linear inflection was 0.86±0.61; the mean Young’s modulus was 18.62±10.30 MPa; and the mean tensile strength was 8.14±5.73 MPa. Hyaluronidase from *Streptomyces hyalurolyticus* had no effect on creep behaviour, whilst chondroitinase ABC prolonged primary creep but had no effect on secondary creep or on any force-extension parameters; it thus appears that neither hyaluronic acid nor sulphated glycosaminoglycans have an interfibrillar load transfer function in the CD. Acetylcholine, the muscarinic agonists arecoline and methacholine, and the nicotinic agonists nicotine and 1-[1-(3,4-dimethyl-phenyl)-ethyl]-piperazine produced an abrupt increase in CD viscosity; the CDs were not differentially sensitive to muscarinic or nicotinic agonists. CDs showed either no, or no consistent, response to adrenaline, L-glutamic acid, 5-hydroxytryptamine and γ-aminobutyric acid. Synthetic echinoid tensilin-like protein had a weak and inconsistent stiffening effect, indicating that, in contrast to holothurian tensilins, the echinoid molecule may not be involved in the regulation of collagenous tissue tensility. We compare in detail the mechanical behaviour of the CD with that of mammalian tendon and highlight its potential as a model system for investigating poorly understood aspects of the ontogeny and phylogeny of vertebrate collagenous tissues.

## Introduction

The feeding apparatus (Aristotle’s lantern) of regular sea-urchins is an integrated complex of skeletal elements, muscles and connective tissue structures. It includes a sub-set of components comprising the compass system, which appears to serve primarily as a respiratory pump whose role is to oxygenate the lantern muscles [1], [2]. This function involves the rhythmic upwards and downwards rotation of five rod-like compass ossicles by the coordinated activity of compass elevators, which are conventional muscles, and compass depressors, which are ligaments consisting mainly of collagen fibres associated with a relatively small population of contractile myocytes [3], [4–6].

The compass depressors (CDs) have attracted considerable attention, because their collagenous component has the capacity to undergo rapid and reversible changes in mechanical properties under physiological control [4–9]. Such mutable collagenous tissue (MCT) is present at several other anatomical locations in sea-urchins [10–16], is ubiquitous in the other extant echinoderm classes and has importance for many aspects of echinoderm biology [17], [18]. As well as representing a phenomenon of great inherent interest, the mechanical adaptability of MCT could provide insight into the pathophysiology, and therefore options for the therapeutic management, of human connective tissue conditions such as disorders affecting the mechanical performance of the uterine cervix and fetal membranes during pregnancy, joint and burn scar contractures, and the weakening of tendons and ligaments due to immobilisation or surgical repair [17]. In addition, MCT is a potential source of inspiration for the development of adaptable materials with biomedical applications. This has already resulted in the development of a polymer nanocomposite with chemoresponsive tensile properties [19], and the feasibility of designing biocompatible materials with site-specific and/or adjustable tensile properties is also being explored using collagen matrices prepared from sea-urchin sources [20], [21].

Although the morphology and biochemistry of the CDs have been thoroughly examined, their mechanical properties and physiology have received less attention [4–9], [22–25]. The aims of the present study were to characterise the static mechanical properties of the CDs of *Paracentrotus lividus* (Lamarck, 1816) by means of creep and incremental force-extension tests and to investigate the effects of: 1) the enzymes chondroitinase ABC and hyaluronidase, in order to evaluate the contribution of glycosaminoglycans to CD tensility, since the mechanical significance of these molecules is a contentious issue in the context of mammalian collagenous tissues [26], [27]; 2) a range of potential neurotransmitter chemicals and their analogues, in order to extend knowledge and better define the basis of the nervous control of CD mutability; and 3) recombinant echinoid tensilin-like protein, in order to assess its potential as an effector molecule involved in the regulation of CD tensility: tensilins are proteins that have been isolated from the MCT of holothurian body wall and that have a stiffening effect on this tissue *in vitro* [28], [29]; we identified a tensilin-like gene in *Strongylocentrotus purpuratus* and synthesised its protein [30], [31].

## Materials and Methods

### Animals and extraction of CD preparations

Specimens of *P*. *lividus* (test diameter 35–65 mm) were collected from the Ligurian coast of Italy at Paraggi (44°18′38″N, 9°12′47″E) and Bergeggi (44°14′37″N, 8°26′40″E) in compliance with national legislation. They were kept in tanks of aerated artificial seawater (ASW: Instant Ocean, Aquarium Systems) at 18°C in the University of Milan and fed with commercial pellets (Wenger Manufacturing, Inc; patent no. 085115204).

**Fig 1:**
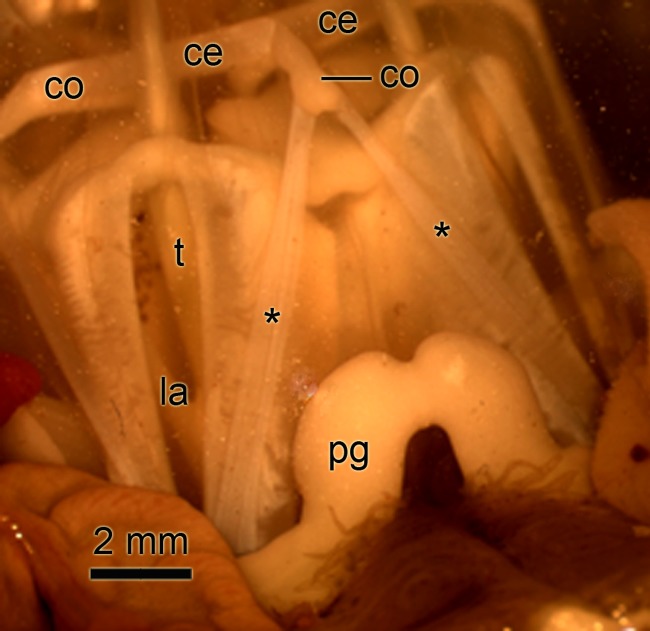
Photograph of the lantern of *Paracentrotus lividus*. The view is from the side and slightly above the lantern. Two of the five segments of the lantern (la) are visible. The compasses (co) are partly elevated due to contraction of the compass elevator muscles (ce). Asterisks, compass depressors; pg, perignathic girdle (edge of test); t, tooth.

**Fig 2:**
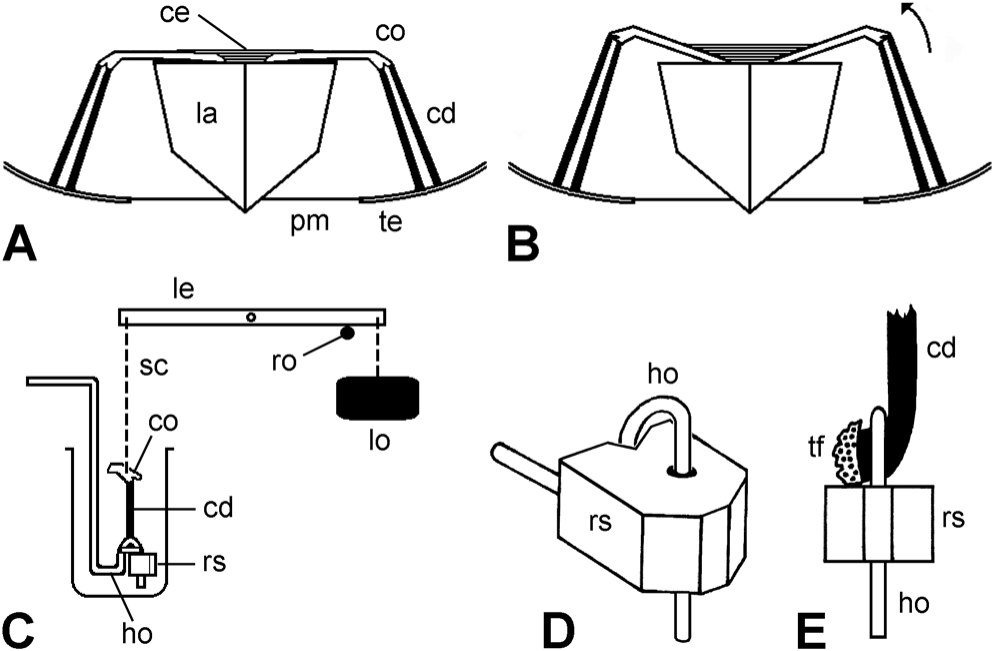
Compass depressors of *P*. *lividus* and experimental set-up; not to scale. (A,B) Diagrammatic lateral views of components associated with the lantern (la) of *P*. *lividus* that are relevant to this investigation. Two compasses (co) and their paired compass depressors (cd) are shown. In A the compasses are fully depressed. Contraction of the compass elevator muscles (ce) effects upwards rotation of the compasses, as shown in B (arrow). pm, peristomial membrane; te, circumoral region of the test. (C) Diagrammatic lateral view of experimental set-up used for creep tests on isolated CDs. ho, hook; le, isotonic lever; lo, load; ro, horizontal rod (shown as transverse section); rs, rubber stop; sc, silver chain. (D) Oblique view of the device designed to grip the test end of each CD. The test fragment to which the CD was attached was trapped in the loop of the wire hook (ho) by a rubber stop (rs) that fitted tightly round the vertical extremity of the hook, but could be slid up and down, as required. (E) Front view of the latter device with a CD in place. tf, test fragment.

To obtain isolated CD preparations, the top half of an animal was removed by cutting through the test with scissors, which exposed the intact feeding apparatus, including the five compass ossicles and five pairs of CDs (Figs. 1, 2A,B). One CD was extracted from each pair together with its skeletal insertion regions, i.e. fragments of the compass ossicle and test to which the CD was attached at its aboral (upper) and oral (lower) ends respectively (Fig. 2A-C). Isolated CDs were kept in ASW or other solution at 14–15°C until needed.

## Mechanical Tests

### Creep tests

**Fig 3:**
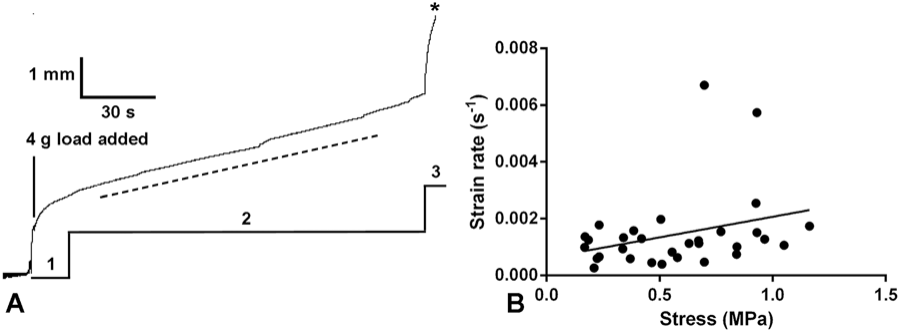
Creep behaviour of *P*. *lividus* CDs. (A) Recording of a representative creep curve, showing the primary (1), secondary (2) and tertiary (3) phases, the last leading to rupture (asterisk). The dashed line indicates the slope of the secondary phase, from which the viscosity was calculated. (B) Relationship between strain rate during the secondary phase and stress. The best-fit line generated by linear regression is included (*y* = 0.0015*x* + 0.0006; Spearman *r* = 0.3076; *P* = 0.0924).

These tests were used to investigate the behaviour of CDs subjected to a constant tensile load. The oral end of each CD was held in the loop of a wire hook by means of an adjustable rubber stop (designed to avoid excessive stress concentration near the attachment point of the CD to the test ossicle fragment; the latter was too fragile to be gripped directly) (Fig. 2D,E). The compass ossicle fragment at the aboral end of the CD was gripped by a heart-clip, which was connected by a silver chain to one end of the lever of an isotonic transducer (Harvard Apparatus Ltd., Edenbridge, England) from the other end of which was suspended a weight. The CD was immersed in a 10 ml bath containing ASW or other solution (Fig. 2C). The weight end of the isotonic lever initially rested on a horizontal glass rod attached to a manipulator. At the start of each test the rod was lowered slowly until the lever was unsupported and the full load was transmitted to the CD. The length of the CD was measured just before the full load was applied. The output from the transducer was fed to a PowerLab 2/26 recorder employing LabChart 7 software (AD Instruments, Oxford, England), which produced a recording of CD extension (change in length) against time (Fig. 3A). Each of the five CDs from each of 14 animals was subjected to a different load (1, 2, 3, 4 or 5 g), in order to determine if there was a relationship between imposed stress and CD strain rate. If a loaded CD had not ruptured after 30 min, the test was terminated. The creep tests, and all other mechanical tests, were conducted at room temperature (16.0–28.5°C).

### Incremental force-extension tests

For these tests the oral end of each CD was linked as above to a wire hook and its aboral end was connected by a heart-clip and silver chain to a force sensor (LCM Systems, Isle of Wight, England). The hook was attached to a manipulator, which enabled the vertical position of the hook and therefore the length of the CD to be adjusted manually by known amounts. At the start of each test, the vertical position of the sensor was adjusted until a force was just recorded; the length of the CD was then measured. The manipulator was then used to lower the hook (thereby extending the CD) in 0.5 mm increments at a predetermined time interval until the CD ruptured; at each increment the hook was lowered at a rate of ca. 1 mm s^-1^, which corresponded to CD strain rates of 0.2–0.7 s^-1^. Each of the five CDs from each of nine animals was extended at a different interval (2, 5, 10, 15 or 20 s, which corresponded to averaged extension rates of 0.25, 0.1, 0.05, 0.033 and 0.025 mm s^-1^ respectively). The output from the force sensor was recorded as above.

### Estimation of cross-sectional area

Data obtained in these experiments were used to calculate nominal stress (force/initial cross-sectional area) and viscosity (nominal stress/strain rate; strain = change in length expressed as a proportion of starting length). The cross-sectional area (CSA) of the CD collagenous component (CDCC) alone (i.e. excluding the peritoneocytes and myocytes of the coelomic epithelia) was estimated from the linear relationship between the mean CDCC CSA and the maximum test diameter (TD): CSA = (0.001662 × TD)– 0.04021 (R^2^ = 0.983). This was obtained by using Adobe Photoshop CS3 to determine the CSA of CDCCs in transverse histological sections of CDs from four animals with test diameters 35, 40, 55 and 65 mm. Measurements were made from 3–5 CDs per animal and 9–15 sections per CD. The sections were 7 μm thick and stained by Milligan’s trichrome method [32]. No attempt was made to measure the CSA of the coelomic epithelial peritoneocytes or myocytes in histological sections, since these components tended to be distorted or incomplete. Estimates of their CSA were based on our previous calculation that in *P*. *lividus* the CDCC occupies approximately (±2%) 86% of the total CD CSA, the myocytes 8% and the peritoneocytes of the coelomic epithelia 6% [5] (Wilkie, unpublished observations).

### Agents tested on CDs

The anaesthetic agent propylene phenoxetol (1-phenoxy-2-propanol) was supplied by Nipa Laboratories, Pontypridd, Wales. All enzymes and neurotransmitter chemicals were purchased from Sigma-Aldrich, Saint Louis, Missouri.

The enzymes were tested on isolated CDs that had been previously immersed in distilled water for 30 min to kill all cellular components. Regarding chondroitinase ABC, CDs were left for 10 h at 37°C in either a solution of the enzyme (0.05 units ml^-1^ Tris-HCl-ASW, pH 8.0), or Tris-HCl-ASW (pH 8.0) alone. They were then subjected to creep tests under a constant load of 5 g or to force-extension tests at an extension rate of 0.5 mm 5 s^-1^. Hyaluronidase (from *Streptomyces hyalurolyticus*) was tested by leaving CDs for 17 h at 37°C in either a solution of the enzyme (100 units ml^-1^ PBS, pH 8.0), or PBS (pH 8.0) alone. They were then subjected to creep tests under a constant load of 5 g.

The neurotransmitters were the cholinergic agonists acetylcholine chloride, methacholine (acetyl-β-methylcholine) chloride, arecoline hydrobromide, nicotine bitartrate and 1-[1-(3,4-dimethyl-phenyl)-ethyl]-piperazine (DPEP), and the non-cholinergic agonists adrenaline bitartrate, γ-aminobutyric acid, 5-hydroxytryptamine and L-glutamic acid. Their effects were investigated by adding them to CDs undergoing creep in ASW. Usually stock solutions were injected by syringe into the organ bath to give desired final concentrations.

Recombinant echinoid tensilin-like protein (TLP) was synthesised as described elsewhere [31]. Briefly, the *S*. *purpuratus* tensilin-like cDNA [30] was used for *in vitro* protein production by means of the EasyXpress Insect Kit II (Qiagen). Proteins were purified with Ni-NTA Magnetic Agarose Beads (Qiagen). The produced protein was dialysed against distilled water to remove the elution buffer and subsequently lyophilised. The produced TLP was quantified by means of the colorimetric and spectrophotometric BCA Assay. To ensure that they were in an initially “soft” (low viscosity) condition, before undergoing creep tests CDs were immersed for 10 min in ASW containing 0.1% propylene phenoxetol (PPASW) [7], [8]. Each CD was then allowed to elongate for 3 min in PPASW under a constant load of 5 g, after which the bath was drained and re-filled with either a solution of TLP (1.5 μg ml^-1^ or 3 μg ml^-1^) in PPASW, or PPASW alone, or PPASW containing the lyophilisate from a volume of dialysed elution buffer equivalent to that in which the TLP had been produced (DEB-PPASW). The latter control was included in case the dialysis process left constituents other than TLP in the elution buffer.

### Comparison of treatments and statistics

The mechanical behaviour of the CDs showed considerable inter-individual variability, probably due, at least in part, to the inherent variability of mutable collagenous structures, which are notoriously resistant to attempts to standardise their physiological state *in vitro*. Unless stated otherwise, when comparing two treatments (e.g. exposure to drug vs. ASW control), two CDs from each experimental animal were subjected to one treatment and the remaining three to the other treatment. Means were calculated from the pooled results for each treatment.

In experiments in which agents were added to CDs undergoing creep, the effect of treatment on CD creep behaviour was quantified as the ‘relative viscosity’, i.e. viscosity during treatment/viscosity before treatment. Since the starting length and the cross-sectional area were the same for both viscosities, and since the extension rate is *inversely* proportional to viscosity, the relative viscosity is equivalent to extension rate before treatment/extension rate during treatment. Unless stated otherwise, the extension rate before treatment was calculated as the mean for the period 60–0 s prior to the start of treatment and extension rate during treatment as the mean for the period 60–120 s after the start of treatment (the latter being chosen to avoid mechanical artefacts caused by the addition of agents).

Statistical analyses were conducted using GraphPad Prism 6. The following parametric tests were applied: Student’s *t*-test (two-tailed), ANOVA with Tukey’s multiple comparison *post hoc* test, and Pearson’s product-moment correlation; the following non-parametric equivalents were applied: the Mann-Whitney *U* test (two-tailed), Kruskal–Wallis test with Dunn’s multiple comparison *post hoc* test, and Spearman’s rank-order correlation. All means are given ± one standard deviation (s.d.).

## Results

### Mechanical properties of compass depressors

Fifty creep tests generated recordings from which data could be obtained. In 21 of these the CD ruptured less than 30 min after the start of loading, thus providing 21 complete creep curves. Rupture occurred usually around the middle of the CDs and not near their attachments to the compass or test ossicle fragments. All other 29 CDs were still extending when the test was discontinued. As observed in many biological materials, the creep curves exhibited three phases: a primary phase in which the extension rate was initially rapid but decreased continuously over a period of up to 2 min; a secondary phase in which the extension rate was constant; and a tertiary phase in which the extension rate increased continuously until the CD ruptured (Fig. 3A). The mean breaking strain, i.e. (length at rupture—starting length)/starting length, was 3.12±1.08 (range 1.41–4.54; N = 9). The primary and secondary phases tended to include transient increases in extension rate after which the rate returned to, or near to, the previous value. The viscosity (i.e. coefficient of viscous traction: nominal stress/strain rate) of each CD was calculated from the slope of the secondary phase of the creep curve (Fig. 3A). The mean viscosity of the CDs was 561±365 MPa.s (range 104–1477 MPa.s; N = 31). Whilst the best-fit line generated by linear regression suggested there might be positive correlation between strain rate and stress (Fig. 3B), this was not statistically significant (Spearman *r* = 0.3076; *P* = 0.0924).

**Fig 4:**
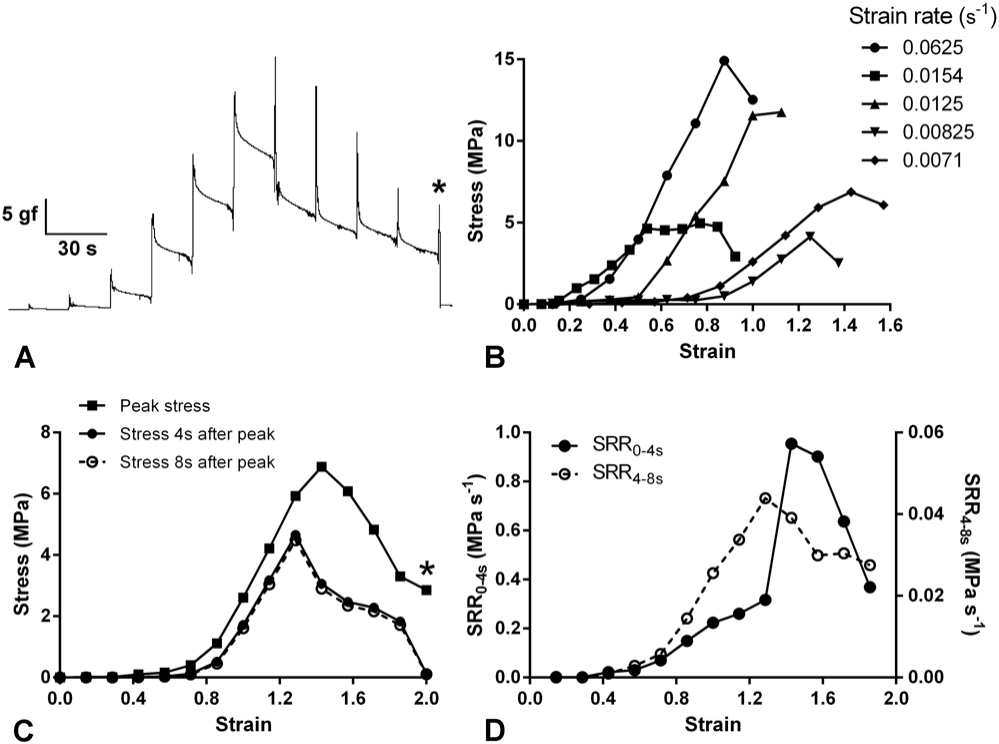
Force-extension and stress relaxation behaviour of *P*. *lividus* CDs. (A) Recording of a representative force-extension test in which the CD was extended by 0.5 mm every 20 s. At each extension the force reached a peak then decayed in two phases (rapid and slow). The asterisk indicates the point at which rupture occurred. (B) Complete set of stress-strain curves derived from force-extension tests on five CDs from one animal. This set of curves is representative in terms of the apparent non-strain rate dependence of the duration of the toe region, the tensile strength and the breaking strain. For the sake of clarity, most of the curves are curtailed before the rupture point. (C) Stress-strain curves of one CD derived from the recording shown in Fig. 4A and which is one of the batch featured in Fig. 4B (strain rate 0.0071 s^-1^). Comparison of the curves for peak stress, stress 4 s after the peak was reached and stress 8 s after the peak was reached. Considerably more stress relaxation occurred in the 4 s after peak stress than in the subsequent 4 s. The asterisk indicates the point at which rupture occurred. (D) Stress relaxation data from Fig. 4C expressed as mean stress relaxation rates (SRR) during the periods 0–4 s and 4–8 s after each peak stress was reached.

Fig. 4A shows the recording of a typical force-extension test. At each 0.5 mm increase in length, the force increased to a peak then fell rapidly. The peak stress-strain curves derived from these recordings were typical for soft biological materials, having an initial toe region during which resistance to imposed strain increased slowly, followed by a steeper region when resistance increased rapidly and became roughly linear, after which a reduction in the slope and an abrupt drop in stress marked the yield point and onset of CD rupture respectively (Fig. 4B). The strain at the inflection between the toe and linear regions was extremely variable (mean 0.86±0.61; range 0.15–3.50; N = 34) (Fig. 4B) and the mean stress at the toe-linear inflection was 1.36±1.37 MPa (range 0.16–6.63 MPa; N = 34); neither inflection parameter showed a significant correlation with the strain rate. The mean tensile strength (maximum stress achieved before breakage) was 8.14±5.73 MPa (range 1.53–23.25 MPa; N = 38). The mean breaking strain (strain at maximum stress) was 1.51±1.07 (range 0.56–6.50; N = 38). There was a weak but statistically significant positive correlation between strain rate and breaking strain (Spearman *r* = 0.4142; *P* = 0.0097) and between strain rate and tensile strength (Spearman *r* = 0.4687; *P* = 0.0030). Stiffness (nominal stress/strain) was calculated as the ‘maximum tangent Young’s modulus’, i.e. the slope of the steepest part of the stress-strain curve for each CD [33]. The mean stiffness was 18.62±10.30 MPa (range 3.31–44.23 MPa; N = 38). Although there was no evidence for a correlation between strain rate and stiffness (Pearson *r* = 0.0569; *P* = 0.7341), there was a positive correlation between the extension rate and stiffness (Pearson *r* = 0.2099; *P* = 0.2059).

The force decay observed after each extensional increment represented the phenomenon known as ‘stress relaxation’. After each increment the initial rate of stress relaxation was high but decelerated within a few seconds to reach a low and almost constant value. This is illustrated in Fig. 4C, which compares the stress 4 s and 8 s after the peak stress was reached. The rates of stress relaxation during the periods 0–4 s after peak stress (“fast stress relaxation”) and 4–8 s after peak stress (“slow stress relaxation”) increased as strain increased, until the strain at which yield (reduction in stiffness) or partial rupture (reduction in peak stress) intervened, after which the stress relaxation rates decreased (Fig. 4D). To obtain measures of the magnitude of CD stress relaxation that could be compared with published information on mammalian tendons, we employed two procedures:

(1) We expressed the reduction in stress occurring 0–10 s after the end of each incremental elongation as a percentage of the peak stress (SR_0–10s_); these SR_0–10s_ values were taken from stress relaxation curves of CDs stretched at a rate of 0.5 mm 15 s^-1^ and 0.5 mm 20 s^-1^ in which 0.5 mm elongation resulted in a constant strain of 0.01–0.10 (to match the range of constant strains associated with published SR_0–10s_ values for mammalian tendons). The mean SR_0–10s_ thus calculated was 95.7% ± 6.8% (range 85.7–100%; N = 7).

(2) We obtained SR_0–10s_ values from stress relaxation curves of CDs stretched at a rate of 0.5 mm 15 s^-1^ and 0.5 mm 20 s^-1^ in which 0.5 mm elongation resulted in a peak stress of 2–3 MPa (to match the peak stress associated with published SR_0–10s_ values for a mammalian tendon). In this case, the mean SR_0–10s_ was 66.8%±18.7% (range 39.3–93.6%; N = 9)

### Effects of enzymes

Pre-treatment with hyaluronidase had no detectable effect on the creep behaviour of the CDs (Table 1). After treatment with chondroitinase, the secondary creep rate (and therefore the viscosity) was unchanged. The duration of, and extension occurring in, the primary creep phase increased significantly, although the mean extension rate (calculated as extension during primary creep/duration of primary creep) during this phase did not change (Table 1).

**Table 1:** Effects of enzymes on the creep behaviour of *P. lividus* CDs.

	Hyaluronidase (6,8)	PBS (5,7)	Chondroitinase (9,9)	Tris-HCl-ASW (8,8)
*Primary creep*				
Extension (mm)	0.21±0.08	0.23±0.07	1.11±0.53^a^	0.47±0.19^a^
Duration (s)	213±120	243±89	594±167^b^	304±215^b^
Extension rate (mm min^-1^)	0.068±0.023	0.060±0.008	0.120±0.057	0.148±0.125
*Secondary creep*				
Extension rate (mm min^-1^)	0.026±0.014	0.022±0.008	0.055±0.028	0.070±0.061

The numbers of values in the primary and secondary creep groups are shown respectively in parentheses. Means sharing the same superscript letter are significantly different (^a^ *P* = 0.001; ^b^ *P* = 0.005; Mann-Whitney tests). There were no other statistically significant differences between enzyme-treated CDs and corresponding control (PBS- or Tris-ASW-treated) CDs.

In view of the influence of chondroitinase on CD creep under constant load, and the ongoing controversy regarding the significance of glycosaminoglycans for collagenous tissue mechanics, the effect of pre-treatment with the enzyme on CD force-extension behaviour was investigated. The enzyme did not change significantly the CD stiffness, tensile strength or breaking strain, nor the strain or stress at which the toe region of the stress-strain curve ended (Table 2).

**Table 2:** Effects of chondroitinase ABC on the force-extension behaviour of *P. lividus* CDs.

	Chondroitinase	Tris-HCl-ASW	*P*
Stiffness (MPa)	29.87±22.24 (9)	39.98±22.99 (8)	0.4731
Tensile strength (MPa)	19.45±5.54 (8)	16.78±5.02 (8)	0.3754
Breaking strain	3.0±2.39 (8)	1.51±1.0 (8)	0.1528
Strain at end of toe region	1.93±1.80 (8)	0.75±0.41(8)	0.1680
Stress at end of toe region	3.72±1.77 (8)	2.24±1.20 (8)	0.0648

Numbers in each experimental group are shown in parentheses. Six animals were used; one or two CDs of each animal were treated with chondroitinase and one or two with medium alone. There were no statistically significant differences between chondroitinase-treated and control groups (probabilities generated by Mann-Whitney tests).

### Effects of neurotransmitters and their analogues

**Fig 5:**
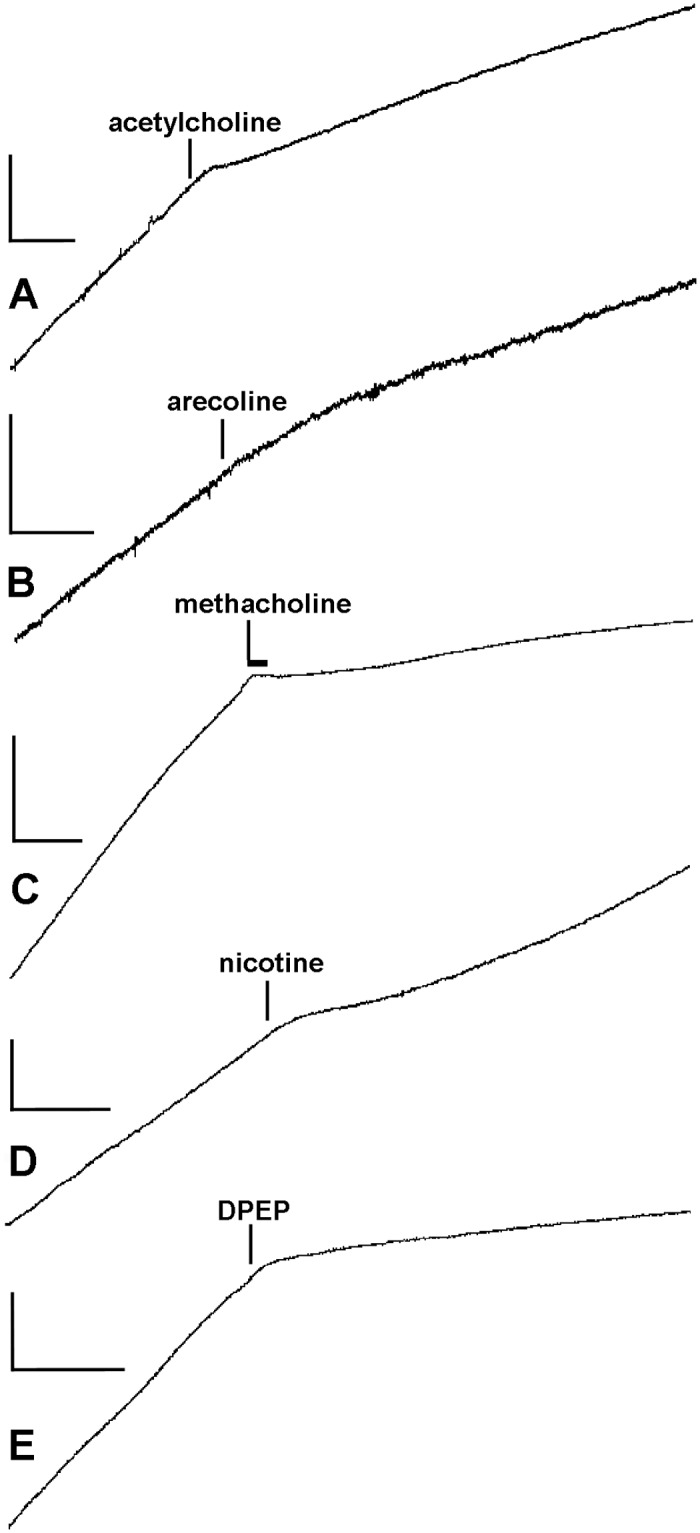
Recordings showing representative effects of cholinergic agonists (all 1 mmol l^-1^). In all cases except C, after the agonist was added it was present to the end of the recording; in C, methacholine was present for only the time period indicated by the horizontal bar, after which it was washed out. Vertical and horizontal scalebars indicate respectively extension and time: (A) 0.2 mm, 60 s; (B) 0.1 mm, 30 s; (C) 0.5 mm, 30 s; (D) 0.2 mm, 30 s; (E) 0.2 mm, 30 s.

The application of acetylcholine and each of the other four cholinergic agonists (all at a concentration of 1 mmol l^-1^) to CDs undergoing creep was followed by a usually abrupt reduction in the extension rate, indicating a sudden increase in viscosity (Fig. 5). Extension never stopped altogether and usually began to increase again (i.e. in the continuing presence of the agonist), though not always reaching the pre-treatment rate before the experiment was terminated (which was 20 min after the start of treatment in the case of acetylcholine, 5 min in the case of the other agents). All recorded responses began within 30 s or less after the start of treatment, only the mean response time for methacholine being significantly different (*viz*. shorter) from that for acetylcholine (Table 3). The magnitudes of the responses, quantified as relative viscosities, are compared in Table 4, which provides no evidence that the CDs are differentially sensitive to muscarinic agonists (arecoline and methacholine) or nicotinic agonists (DPEP and nicotine). Because the response of two of the nicotine-treated CDs decayed rapidly (Fig. 5D), the difference between the mean relative viscosities of nicotine-treated and control CDs was not statistically significant when these were calculated as usual using mean extension rates 60–120 s after the start of treatment; the difference was significant when mean extension rates 0–60 s after the start of treatment were used (Table 4).

**Table 3:** Response times (delay between addition of agent and start of response) of *P. lividus* CDs treated with cholinergic agonists.

	Response time (s)			
	Mean	s.d.	Range	N
Acetylcholine	11.7	5.8	6–24	11
Arecoline	12.7	12.0	4–30	6
DPEP	12.2	8.5	4–25	5
Methacholine	2.7	1.6	1–6	7
Nicotine	5.4	4.0	2–11	5

Only the methacholine response time differed significantly from that for acetylcholine (*P* = 0.01; Kruskal-Wallis and Dunn’s tests). DPEP, 1-[1-(3,4-dimethyl-phenyl)-ethyl]-piperazine.

**Table 4:** Effects of pharmacological agents on the relative viscosity of *P*. *lividus* CDs.

	Relative viscosity			
Agent	Agonist (N)	Control (N)	Agonist/Control	*P*
*Cholinergic agonists*				
Acetylcholine	3.09±1.15 (21)	1.62±0.51 (19)	1.9	<0.0001
Arecoline	1.98±0.36 (10)	1.50±0.29 (9)	1.3	0.0053
DPEP	4.29±1.31 (5)	1.28±0.29 (5)	3.3	0.0079
Methacholine	8.45±7.39 (7)	1.87±0.91 (6)	4.5	0.0012
Nicotine_60–120s_	6.21±5.88 (4)	1.28±0.90 (5)	4.8	0.0635
Nicotine_0–60s_	4.77±2.87 (4)	1.18±0.41 (5)	4.0	0.0159
*Other agonists*				
Adrenaline	0.75±0.36 (4)	0.66±0.59 (3)	-	0.8252
γ-Aminobutyric acid	2.56±1.93 (5)	1.50±0.31 (5)	-	0.9444
L-Glutamic acid	1.02±0.81 (5)	1.47±0.25 (4)	-	0.3262
5-Hydroxytryptamine	1.81±0.59 (5)	1.20±0.38 (4)	-	0.1180

No change in extension rate was discernible after the application of 1 mmol l^-1^ adrenaline, L-glutamic acid or 5-hydroxytryptamine (Table 4). The same concentration of γ–aminobutyric acid had no consistent effect (Table 4): its application was followed by no discernible change in the extension rate (one CD), a transient increase (one CD), a transient decrease (one CD) and a sustained decrease (two CDs).

The two-tailed probability values were generated by Student’s *t* and Mann-Whitney *U* tests. In all controls, ASW alone was added to the preparation. ‘Relative viscosity’ (viscosity during treatment/viscosity before treatment) was calculated from the mean extension rates during the periods 60–120 s after the start of treatment (addition of neurotransmitter or ASW alone) and 60–0 s before the start of treatment, except for nicotine_0–60s_, in which case ‘viscosity during treatment’ was calculated from the mean extension rate 0–60 s after the start of treatment. Comparison of the relative effects of the cholinergic agonists (standardised as mean relative viscosity of treated CDs/mean relative viscosity of control CDs: see column *Agonist/Control*) suggests that the CDs were not differentially sensitive to muscarinic agonists (arecoline and methacholine) or nicotinic agonists (DPEP and nicotine).

### Effects of recombinant echinoid tensilin-like protein

Before conducting tests with synthetic tensilin-like protein (TLP), we confirmed that PPASW lowered CD viscosity by adding either PPASW or ASW alone to CDs extending under a constant load of 5 g. The mean relative viscosity of the PPASW-treated CDs 180–240 s after the start of treatment was 0.91±0.88 (N = 11) and that of the ASW-treated controls was 1.66±0.42 (N = 11), which represented a significant difference (*P* = 0.0033; Mann-Whitney test).

**Fig 6:**
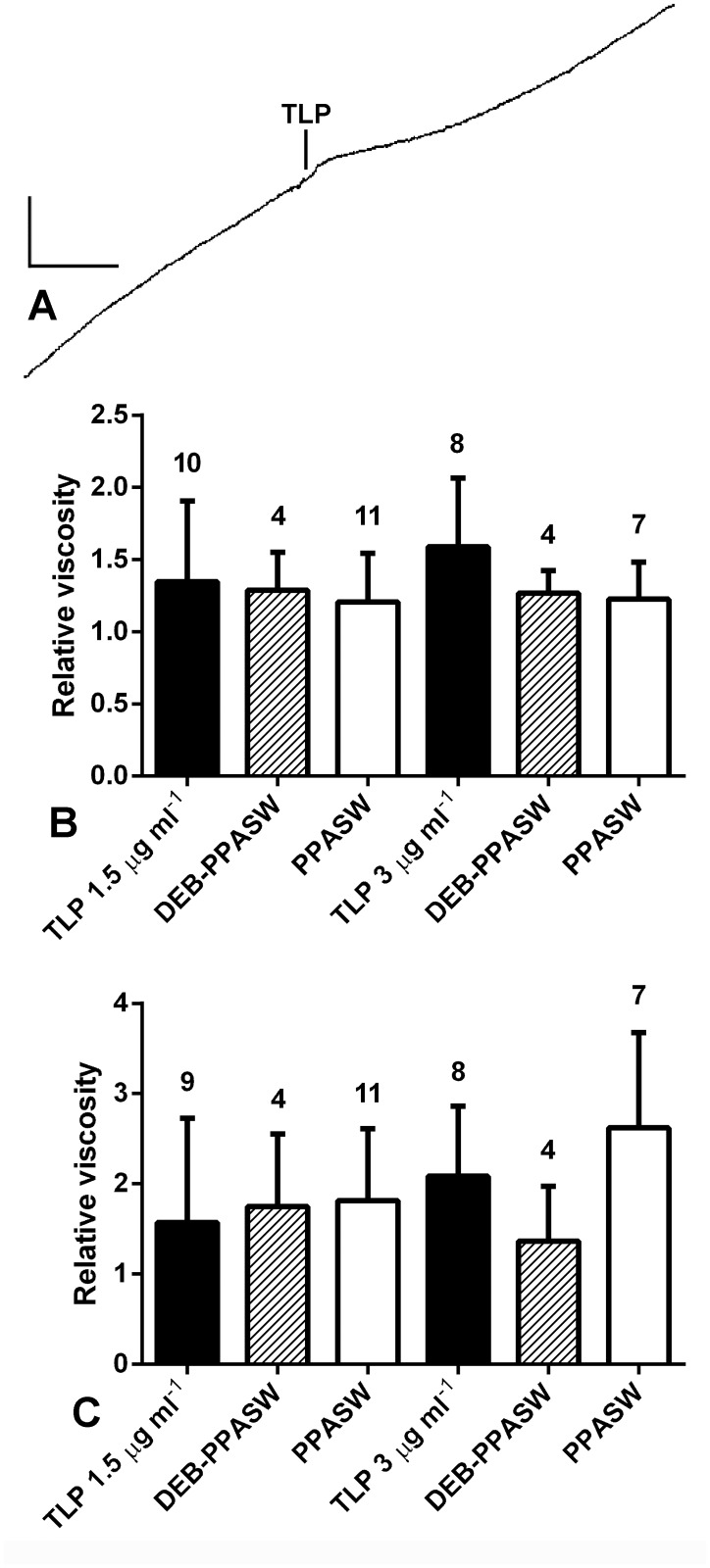
Effect of tensilin-like protein (TLP) on the creep behaviour of *P*. *lividus* CDs. (A) Recording of a response to 3.0 μg ml^-1^ TLP; vertical scalebar, 0.2 mm; horizontal scalebar, 60 s. (B, C) Effect of TLP on relative viscosity. CDs were treated with TLP at concentrations of 1.5 μg ml^-1^ and 3.0 μg ml^-1^; separate DEB-PPASW and PPASW controls were used for each concentration. (B) Mean relative viscosities for the time period 15–75 s after the start of TLP treatment. (C) Mean relative viscosities for the time period 5–6 min after the start of TLP treatment. Error bars are standard deviations and numbers of CDs in each group are shown above error bars.

The application of TLP to PPASW-treated preparations elongating under a constant load was followed by an abrupt reduction in the extension rate of three out of the ten CDs treated with 1.5 μg ml^-1^ TLP and two out of the eight CDs treated with 3 μg ml^-1^ TLP; these responses started 6–20 s after the start of TLP treatment and in three cases the extension began to increase again after 40–50 s (Fig. 6A). No abrupt changes in extension rate were seen in the controls (addition of DEB-PPASW or PPASW alone). To determine if TLP produced a more gradual decrease in the extension rate of those CDs that did not show an abrupt change, the relative viscosity 15–75 s after the start of TLP treatment was compared with that of the control groups. The mean relative viscosity after treatment with 3 μg ml^-1^ TLP started was higher than that after 1.5 μg ml^-1^ TLP and both were higher than those of control CDs treated with DEB-PPASW or PPASW alone (Fig. 6B). However, the differences between TLP-treated and control CDs did not reach statistical significance (*P* = 0.5502; Kruskal-Wallis test). To determine if TLP had any longer term action, mean relative viscosities for the time period 5–6 min after treatment started were compared. This provided no evidence for a stiffening effect (*P* = 0.1259; Kruskal-Wallis test) but showed that the behaviour of all treatment groups, most noticeably the controls, was more variable than at 15–75 s, as indicated by their larger standard deviations (Fig. 6C).

## Discussion

### Morphological and microstructural background

**Fig 7:**
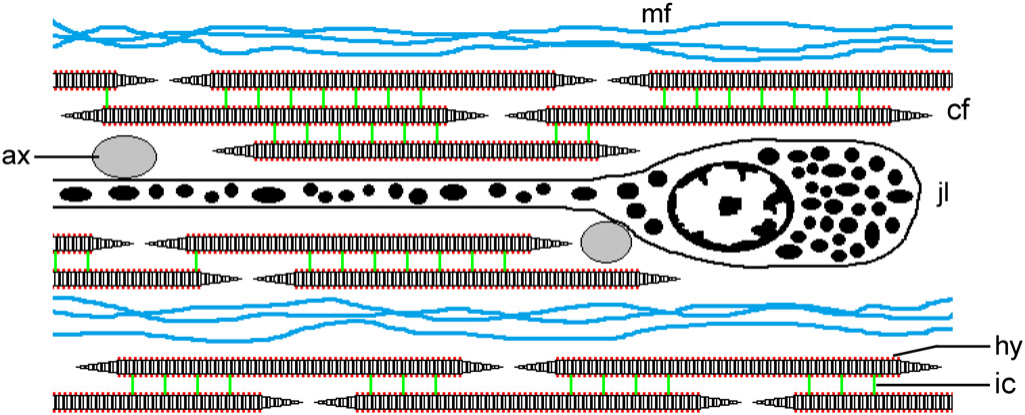
Diagrammatic representation of the CD of *P*. *lividus*. This depicts part of a longitudinal section; not to scale. The CD consists of parallel discontinuous collagen fibrils (cf), which are connected by interfibrillar crossbridges containing chondroitin sulphate/dermatan sulphate proteoglycans (ic). Hyaluronic acid (hy) is present but of unknown function (its disposition on the surface of the collagen fibrils is conjectural). Bundles of beaded microfibrils (mf) occur between the collagen fibrils. The main cellular components are the somata and processes of electron-dense granule-containing juxtaligamental cells (jlc). Agranular cell processes, which may be cholinergic axons, are in close contact with the juxtaligamental cells and are shown as transverse sections (ax).

Each CD of camarodont echinoids like *P*. *lividus* is a strap-shaped collagenous ligament enclosed within its own coelomic canal and ensheathed by coelomic epithelia that separate it from the adjacent fluid compartments. The ligament consists mainly of bundles of striated collagen fibrils, which are parallel to the longitudinal axis of the CD. Cellular elements within the ligament include juxtaligamental somata and processes, which probably regulate the tensile properties of the extracellular matrix, and neural processes [4], [5], [7], [34] (Fig. 7). The coelomic epithelium on the outer (abaxial) surface of the ligament comprises only peritoneocytes; that on the inner (adaxial) surface is a pseudostratified myoepithelium comprising peritoneocytes and elongated myocytes arranged parallel to the collagen fibres (= fibril bundles) [34].

The collagen fibrils of the CD contain type I collagen, have on their outer surface periodically distributed sulphated chondroitin sulphate/dermatan sulphate-like glycosaminoglycans [7], [8] and are interspersed with a smaller population of longitudinally arranged beaded microfibrils resembling those found to contain fibrillin in another MCT [5], [35] (Fig. 7). The CD extracellular matrix thus resembles that of mammalian tendon, in that the latter also consists mainly of bundles of parallel type I collagen fibrils with periodically distributed sulphated glycosaminoglycans and associated fibrillin-containing microfibrils [36], [37]. Furthermore, as will be discussed below, the mechanical properties of the CD indicate clearly that its collagen fibrils are discontinuous, i.e. they are shorter than the total length of the CD, and the weight of evidence suggests that this is also the case in mammalian tendon [38–40]. The contrast between the similar microstructural organisations of the CD and mammalian tendon on the one hand and their differing mechanical properties on the other will be a theme of the following discussion.

### Mechanical properties

#### Physiological condition of isolated CDs

Being mutable collagenous structures, the CDs exhibit a wide range of viscosity and stiffness [4], [5], [7–9]. Our experiments were conducted mostly on *P*. *lividus* CDs that, after excision from the host animal, were held in ASW without intervening or further treatment. Experimental manipulation of such CDs can switch them to a state of lower mechanical resistance (= “soft”) or higher mechanical resistance (= “stiff”) [4], [5], [41]. We therefore consider recently isolated and untreated CDs as being in a “baseline” physiological condition, which may be equivalent to the “standard” state of Motokawa and co-workers’ three-state model of holothurian dermis [29], [42], [43]. However, in view of the variability of the mechanical parameters exhibited by CDs in the baseline condition (see below), it is better described as an intermediate range of mechanical resistance rather than as a narrowly delimited tensile state.

#### Contribution of non-collagenous components to CD mechanical properties

We assumed that the forces recorded in our experiments resulted overwhelmingly from the passive mechanical resistance of the CD collagenous component and that the contribution of the non-collagenous components (peritoneocytes and myocytes of the coelomic epithelia) was negligible, for the following reasons. The peritoneocytes (which occupy ca. 6% of the total CD CSA) possess no cytological features (e.g. tonofilament aggregations, prominent or abundant desmosomes) that suggest they have a stress transfer function [5] (Wilkie, unpublished observations). The possible contribution of the myocytes (which occupy 8% of the total CD CSA) was assessed indirectly using what appear to be the only published values for the tensile strength of an echinoderm muscle. The longitudinal body wall muscle (LBWM) of the holothurian *Holothuria tubulosa* was found to have a tensile strength of 0.059±0.023 MPa when relaxed and 0.206±0.080 MPa when contracted [44]. Assuming the CD myocytes had the same tensile strength, we calculated the force needed to break the myocyte component of each CD (breaking force = tensile strength × CSA [estimated from the CSA of the collagenous component]) and expressed this as a proportion of the recorded breaking force. The myocyte breaking force was thus estimated to be on average 0.1±0.0008% or 0.4±0.0029% (N = 38) of the recorded force (based on the relaxed and contracted LBWM breaking strength values respectively). Whilst we acknowledge that this refers to only one parameter and that the actual mechanical properties of the CD myocyte component are unknown, we regard this calculation as being a strong indication that in general it is highly unlikely that the relative contribution of the myocytes to the passive mechanical properties of the CD exceeded the margins of error associated with our quantitative analysis. In the following discussion it is therefore assumed that the values of all mechanical parameters (i.e. viscosity, stiffness, tensile strength etc.) apply to the collagenous component alone.

#### Creep tests

The creep curves of CDs subjected to a constant load demonstrated a primary phase during which extension was initially rapid then decelerated, a secondary (or minimum creep rate) phase during which the extension rate remained steady, and a tertiary phase during which the extension rate accelerated until rupture occurred. Similar three-phase creep curves have been observed in all investigated echinoderm collagenous structures [45] and in mammalian collagenous tissues [46], although the minimum creep rate phase of certain mammalian tendons occurs only as the inflection between the primary and tertiary phases [47]. The primary phase of mammalian structures has been attributed to either the straightening out of initially wavy or crimped collagen fibres (i.e. fibril bundles) in the case of parallel-fibred structures like tendons and ligaments [47], [48], or to the progressive pulling into alignment of collagen fibres along the loading axis in the case of three-dimensional networks like dermis [49]. In both these cases the load is progressively transferred to the collagen fibres and the start of the secondary phase (or, where there is no secondary phase, the inflection between the primary and tertiary phases) marks the point at which the fibres bear the full load. The primary phase of *P*. *lividus* CDs probably results from the straightening out of the fibril bundles, which tend to have a wavy appearance [7].

The secondary phase of the creep curves of parallel-fibred mutable collagenous structures results from the lengthening under tension of the collagen fibres (fibril bundles) through the shearing past each other of their constituent fibrils, which are discontinuous, i.e. shorter than the total length of the structure [50–52]. This was demonstrated clearly by the CDs, since they elongated under stresses that were at least two orders of magnitude lower than the tensile strength of individual echinoderm collagen fibrils and CD breaking strains were around four times those of individual fibrils (S1 Table and S2 Table) [53], [54]. The constant strain rate that characterises the secondary phase is due to the continuous disruption *and replacement* of unstable, non-covalent linkages between adjacent collagen fibrils as they slide over each other. The coefficient of viscosity (stress/secondary phase strain rate) of the CDs was of the same order of magnitude as those of other echinoderm parallel-fibred mutable collagenous structures (echinoid central spine ligament and ophiuroid intervertebral ligament), but more than three orders of magnitude less than that of wallaby tail tendon (S1 Table). The CDs also differed from mammalian tendon in exhibiting much greater breaking strains (S1 Table). In this investigation all CDs ruptured within 30 min after the start of the test or were continuing to extend when tests were terminated after 30 min, which contrasts with our previous finding that *P*. *lividus* CDs under constant load extended for 1–5 min then showed no further lengthening, i.e. they exhibited a creep limit [5]. The discrepancy is probably due to the different stresses that were applied: around 60 kPa in the earlier experiments and 168–1163 kPa in the recent experiments, and it implies that the applied stress has to exceed a threshold (between 60 and 160 kPa) before secondary creep is initiated. An analogous situation was observed in wallaby tail tendon, which exhibited creep when subjected to a stress of 20 MPa or more, but not at 10 MPa [47]. The authors estimated that the *in vivo* stress on this tendon was ca. 14 MPa when its muscle contracted isometrically and therefore around the stress threshold above which damage (due to creep) might be caused. Because the length of CDs at the creep limit is the same as the maximum length attained by CDs during the activities of intact lanterns [5], it is likely that the stresses at which a creep limit is observed are within the range encountered by the CDs *in vivo* and that stresses that produce secondary creep exceed that range and are non-physiological.

Despite a suggestive regression line, there was not a significant correlation between the strain rate during secondary creep and the constant stress. This is anomalous. For example, in another mutable collagenous structure (ophiuroid intervertebral ligament: [55]) and in mammalian tendon [47], [56], the creep rate increases with stress, as would be expected, since, the greater the stress, the greater the rate at which linkages responsible for tissue cohesion are broken. We suspect that our failure to demonstrate such a relationship in the CD was due to the masking effect of intra-individual variability in the CD starting length: the ratio of the longest to the shortest starting lengths per individual ranged from 1.32 to 3.05.

#### Incremental force-extension tests

The peak stress-strain curves, which were typical for collagenous structures (mammalian tendon: [57], [58]; other echinoderm mutable collagenous structures: [59–61]), comprised a low resistance toe region, which in other structures is attributed mainly to straightening of collagen fibres, a steeper roughly linear region resulting from the direct loading of the fibres, and a yield region (indicated by a reduction in the slope of the curve) caused by initial microscopic damage (limited fibril or fibre breakage), which ended suddenly with complete rupture or was followed by a more or less rapid and sometimes stepped decline in stress (caused by progressive macroscopic damage) leading to complete rupture [48], [58–61]. The strain at which the linear region started was very variable (mean 0.86, range 0.15–3.50), but was one to two orders of magnitude greater than the equivalent for mammalian tendon (ca. 0.02: [57], [58]. Likewise, the CD breaking strain (mean 1.51, range 0.56–6.50) was much greater than that reported for mammalian tendon (ca. 0.10: S2 Table). The stress at the toe-linear inflection ranged from 0.16 to 6.63 MPa, which, if our inference that the CD is not subjected to tensile stresses greater than ca. 0.1 MPa *in vivo* is correct (see above), implies that the CD is not strained beyond the toe region of its stress-strain curve *in vivo*.

The tensile strength and stiffness of the CDs were similar to those of other parallel-fibred mutable collagenous structures (echinoid spine ligament and ophiuroid intervertebral ligament: S2 Table), but, in comparison with values reported for mammalian tendon, the tensile strength was around one order of magnitude lower and the stiffness around two orders of magnitude lower (S2 Table). In certain mutable collagenous structures [51], [60] and in mammalian tendons [57], [62], [63], tensile strength and stiffness increase with increasing strain rate. The tensile strength of the CDs showed such strain-rate dependence, but the stiffness did not. While the stiffness and other mechanical properties of another MCT—the body wall of the starfish *Coscinasterias calamaria—*also appear to lack strain-rate dependence [61], its microstructural organisation is much more complex than that of the CD. It is possible that CD stiffness is strain-rate dependent, but that this relationship was obscured by intra-individual variability of CD starting lengths (which were used to calculate strain). It should be noted that the values for tensile strength and stiffness given in this paper differ by an order of magnitude from those published previously [4], [5], which are incorrect due to the miscalculation of cross-sectional areas.

The asymptotic pattern of stress relaxation displayed by CDs, i.e. an initial rapid loss of stress decelerating quickly to a low and almost constant rate, has been observed in all soft collagenous structures subjected to constant strain [40], [59], [64–66] and is due initially to collagen fibrils and fibril bundles shearing past each other under uniaxial tension (fast stress relaxation), followed by the slower breakage of interfibrillar linkages (slow stress relaxation) [40], [67], [68]. In mammalian tendon the rate of stress relaxation increases with increasing constant strain [66], [69], [70]. This relationship was also demonstrated clearly by CDs: the rates of both fast and slow stress relaxation (measured during the 0–4s and 4–8s respectively after each 0.5 mm lengthening) increased steadily with increasing strain until yield or complete rupture occurred. CDs in which the first 0.5 mm elongation imposed a strain of 0.06–0.10 showed a stress relaxation of 86–100% (mean 95.7% ± 6.8%) 0–10s after elongation stopped; the equivalent measure of stress relaxation (SR_0–10s_) for three different mammalian tendons subjected to constant strains of 0.02–0.075 was 9–50% (mean 25.7% ± 14.4%; see S3 Table), which demonstrates that at a constant strain of ca. 0.05 CDs undergo significantly more fast stress relaxation than do mammalian tendons (*P* = 0.0006; Mann-Whitney test). Similarly, the mean SR_0–10s_ of CDs after peak stresses of 2–3 MPa was 66.81%±18.71%, which is considerably greater than the 25–33% shown by porcine digital flexor tendon after a peak stress of 2 MPa [70].

### Effects of enzymes

All collagenous tissues, including echinoderm MCTs, contain glycosaminoglycans (GAGs), most of which occur as sidechains attached to the protein core of proteoglycan (PG) molecules [17], [71]. Some of the PGs present in mammalian collagenous tissues and echinoderm MCTs are located on the surface of the collagen fibrils and are components of structures that, in the transmission electron microscope, appear to form bridges between adjacent fibrils [52], [72]. These observations contributed to the idea that, when these tissues are stretched, PGs transfer load between collagen fibrils and thereby contribute significantly to tissue extension mechanics [73]. Whilst considerations of chemical morphology and biophysics provide a plausible theoretical basis for PGs having such a force transfer function [26], there is empirical evidence both for and against this being the case in mammalian collagenous tissues [27], [74–78].

The extracellular matrix of *P*. *lividus* CDs contains a diversity of GAGs, including hyaluronic acid and chondroitin sulphate/dermatan sulphate, the latter forming bridge-like structures disposed periodically on the surface of the collagen fibrils [7], [25] (Barbaglio, unpublished data) (Fig. 7). We investigated the possible contribution of these particular GAGs to CD mechanics by treating CDs with hyaluronidase from *Streptomyces hyalurolyticus*, which degrades only hyaluronic acid, and with chondroitinase ABC, which degrades dermatan sulphate, chondroitin sulphate and hyaluronic acid (the last only slowly) [79], [80]. Hyaluronidase had no detectable effect on creep behaviour, suggesting that hyaluronic acid is not involved directly in interfibrillar linkage. Chondroitinase prolonged primary creep, an indication that sulphated GAGs may inhibit the complete straightening of collagen fibres during this phase, but had no effect on secondary creep or on any force-extension parameters. It thus appears that, since their removal did not change CD behaviour at strains where the collagen fibrils were directly loaded (i.e. in the secondary creep phase and in the linear region of the stress-strain response), sulphated GAGs do not have a load transfer function in this tissue. In accord with these findings, hyaluronidase from ovine testes, which digests hyaluronic acid, chondroitin and chondroitin sulphate, did not affect the viscosity of an ophiuroid intervertebral ligament, although this was shown by histochemical methods to contain hyaluronidase-labile GAGs [55]. On the other hand, Junqueira and co-workers found that the thiol proteinase papain caused “softening” of holothurian dermis and the splitting apart of collagen fibres into separate fibrils [81], suggesting that in at least some collagenous tissues the protein core of PG molecules might be more important for interfibrillar cohesion than are the GAG sidechains. This can also be inferred from the finding that the association between PG and the collagen fibrils of the echinoid spine ligament depends on the core protein and not on its chondroitinase ABC-removable GAG [52], and would explain the insensitivity of the tensile properties of some mammalian collagenous tissues to GAG depletion [27], [82].

### Effects of neurotransmitters

Our aim in testing the effects on the CD of a range of neurotransmitters was limited to screening for responsiveness rather than to conduct a pharmacokinetic comparison. Acetylcholine and four other cholinergic agonists produced an abrupt increase in the viscosity of CDs undergoing creep. There was little evidence that the CDs show consistent differences in their responses to muscarinic and nicotinic agonists: although the response time to one muscarinic cholinomimetic (methacholine) was significantly shorter than that to acetylcholine, there was no significant difference in the magnitudes of the responses (measure as relative viscosities). The CD of *P*. *lividus* contains a diversity of axon-like cell processes, including a large diameter agranular type that is in close contact with the juxtaligamental cells and which may be cholinergic (Fig. 7). Other granule-containing processes associated with the coelomic epithelia suggest that aminergic or some other type of non-cholinergic neurotransmission might also be involved in the control of CD mechanics [4], [5]. The absence of a significant response to any of the non-cholinergic neurotransmitters was therefore disappointing and indicates that a wider range of agonists should be investigated.

It needs to be emphasised that the responses to cholinergic agonists resulted predominantly from a passive increase in the viscosity of the collagenous component of the CD and that active contraction of the myocytes would have made little, if any, contribution. We estimated the contractile stress that the myocytes would have had to generate if they alone had been responsible for the neurotransmitter-induced decrease in extension rates observed in our experiments. To slow or arrest creep, the myocytes would have had to exert a force at least as great as that imposed by the constant load. Stress was calculated as constant load/myocyte CSA, the latter being estimated from the collagenous component CSA, assuming that the collagenous component and the myocytes occupy 86% and 8% of the total CD CSA respectively. The minimal myocyte contractile stress was 6.6 MPa for acetylcholine-induced responses; 7.8 MPa for arecoline; 7.4 MPa for methacholine; 7.6 MPa for nicotine; and 7.3 MPa for DPEP (constant load 4 g in all cases). In other tests in which the constant load was 8 g (not included in the quantitative results given in this paper) and in which 1 mmol l^-1^ methacholine completely arrested extension, the myocytes would have had to generate a minimal stress of 15.8 MPa. Bearing in mind that the strongest contraction reported for any muscle is 1.34 MPa (*Mytilus* anterior byssus retractor muscle: [83]) and that the maximal contractions recorded for two echinoderm muscles were 160 kPa (*Anthocidaris crassispina* spine muscle: [84]) and 63 kPa (*Holothuria tubulosa* longitudinal body wall muscle: [44]), it is highly unlikely that the myocytes had any role in the responses to cholinergic agonists.

Our results provide further evidence for the importance of cholinergic pathways in the control of MCT tensile properties. These pathways are believed to include the motor input that regulates the activities of the juxtaligamental cells. There is ultrastructural evidence for functional links between cholinergic axons and the juxtaligamental cells of echinoid and ophiuroid ligaments [85], [86]. Almost all investigated mutable collagenous structures have been found to be sensitive to exogenous acetylcholine, although its effect is variable: it reduces the mechanical resistance (measured as stiffness or viscosity) of crinoid MCT (see e.g. [87]), whereas in the other four echinoderm classes it increases mechanical resistance (occasionally having a biphasic effect: increase then decrease) (see e.g. [16], [60], [88], [89]). Like the CD, other echinoderm mutable collagenous structures are usually responsive to both muscarinic and nicotinic agonists [90–92], though an exception is a starfish body wall preparation, which was affected only by muscarinic agonists [93]. Whilst the muscarinic and nicotinic responses of most MCT preparations differ quantitatively rather than qualitatively, nicotinic receptors seem to mediate an increase in the viscosity of holothurian dermis and muscarinic receptors a decrease in its viscosity [88]. It is thus evident, despite the patchiness of current information, that cholinergic mechanisms associated with the control of MCT tensile properties show a surprising diversity that appears to be correlated with taxonomic class and therefore betokens a long evolutionary history.

### Effects of echinoid tensilin-like protein

**Fig 8:**
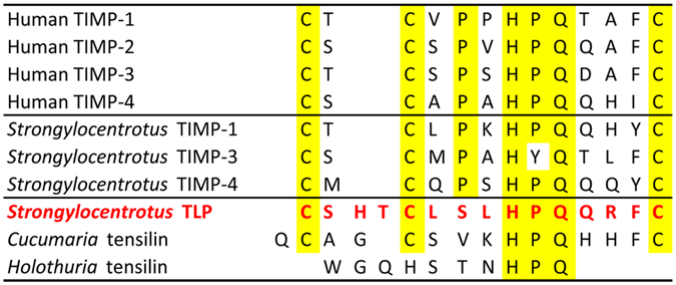
Comparison of amino acid sequences. Alignment of the amino acid sequences of the immediate N-terminal domains of human and sea-urchin TIMPs, holothurian tensilins and *Strongylocentrotus* tensilin-like protein. Identical amino acids are highlighted. (Sources: [28–30], [95], [107]).

*C-*tensilin and *H*-tensilin are proteins that have been isolated from the dermis of two holothurian species belonging to different taxonomic orders and which both stiffen the dermis *in vitro* and aggregate isolated holothurian collagen fibrils. *C-*tensilin has been immunolocalised to the neurosecretory-like juxtaligamental cells of the dermis and both proteins are believed to be regulatory factors involved in the control of dermal stiffness by participating directly in interfibrillar linkage formation [28], [29], [94]. Furthermore, holothurian tensilins and echinoid tensilin-like protein (TLP) have significant similarity to the tissue inhibitor of metalloproteinase (TIMP) family of proteins, particularly at their N-terminal domains, which in TIMPs are responsible for the matrix metalloproteinase (MMP) inhibitory activity (Fig. 8) [30], [95]. This similarity provoked the suggestion that the echinoid TLP might also contribute indirectly to CD stiffening *in vivo* by inhibiting MMP-dependent interfibrillar crosslink degradation [9].

The stiffening effect of holothurian tensilins is limited: holothurian dermis seems to have three mechanical states (“soft”, “standard”, “stiff”) and tensilins can convert it from the “soft” to the “standard” state but not from the “standard” to the “stiff” state [29], [43]. We therefore tested synthetic echinoid TLP on CDs that were in a soft (i.e. low viscosity) condition due to pre-treatment with PPASW [7–9]. We found that TLP at a concentration of 1.5 μg ml^-1^ or 3 μg ml^-1^ caused at best a weak, inconsistent and transient increase in the viscosity of the CD, which provides little support for the possibility that this protein contributes directly or indirectly to interfibrillar linkage in the CD *in vivo*. Bearing in mind that mammalian TIMPs are themselves multifunctional proteins with activities unrelated to MMP inhibition [95], it is possible that, though belonging to the TIMP family, echinoid TLP is neither a MMP inhibitor nor a participant in MCT physiology. The former is probable, because the first and second cysteine residues of the functionally critical N-domains of mammalian and echinoid (*Strongylocentrotus*) TIMPs are separated by a single amino acid, whereas in TLP these cysteines are separated by three amino acids, which may alter its tertiary structure (Fig. 8) [30], [95]. We applied TLP at concentrations similar to those of *H*-tensilin that stiffen holothurian dermis *in vitro* (1–3 μg ml^-1^: [29]). Resource limitations prevented us from conducting a proper dose-response investigation, which would have revealed if the CDs are more responsive to higher TLP concentrations.

## Conclusions

Although in both the creep and incremental force-extension tests the CDs were subjected to a range of stresses that extended well above the inferred *in vivo* limit, our results, like those derived from non-physiological investigations of mammalian tissues (see e.g. [47]), provide an insight into the properties of the CD as a material. Like many mammalian tendons, the CD demonstrated (1) a three-phase creep curve under constant loads (above a certain threshold), (2) a three-phase stress-strain curve most of the parameters of which exhibited strain rate dependence and (3) rapid then slow phases of stress relaxation when held at a constant strain, with stress relaxation rates increasing as constant strain increased. However, in comparison with the equivalent values for mammalian tendon, the coefficient of viscosity of the CD was more than three orders of magnitude lower, its tensile strength was an order of magnitude lower, its stiffness was two orders of magnitude lower, and it showed two to three times more stress relaxation (S1–S3 Tables). The mechanical properties of the CD and mammalian tendon are thus qualitatively similar but quantitatively very different. Both structures consist of parallel arrangements of discontinuous type I collagen fibrils between which is a complex chemical environment that includes entities responsible for interfibrillar cohesion. Mammalian and echinoderm collagen fibrils have a similar tensile strength and Young’s modulus (S2 Table). This by itself would not exclude the possibility that the collagen fibrils contribute to the dissimilarities in the mechanical properties of their native tissues, because differences in other fibril attributes, such as length, diameter, aspect ratio and packing density, could be at least partly responsible [52], [75]. Nevertheless, differences in the properties of their collagen fibrils could not explain the key distinguishing feature of the CDs (and other echinoderm MCTs), which is their ability to change their mechanical resistance drastically within a timescale of seconds: for example, when appropriately stimulated the parallel-fibred echinoid spine ligament can undergo a 10-fold increase in elastic modulus, which brings it within the stiffness range shown by mammalian tendons (S2 Table); and the tensile strength of the CDs of *P*. *lividus* increases 20-fold (from 3.4 MPa to 66.7 MPa, the latter again being within the range for mammalian tendon: S2 Table; calculated from data in [41]) when treated with distilled water. The variable tensility of echinoderm MCTs can be due only to changes in the linkages holding together adjacent collagen fibrils that determine the degree to which the linkages resist or permit interfibrillar shear.

Independent evidence demonstrates clearly that there is a major difference in the properties of the interfibrillar linkages of MCT and vertebrate collagenous tissues. Whereas the disaggregation of MCT (including *P*. *lividus* CDs) and extraction of intact isolated collagen fibrils can be achieved by mild chemical and mechanical methods [25], [96], [97], it has proved very difficult to extract isolated collagen fibrils from normal adult vertebrate collagenous tissues [98–100]. MCT is therefore distinguished by the labile nature of its interfibrillar linkages (which can, however, switch reversibly to a mechanically stable state), in contrast to the permanently stable linkages of adult vertebrate tissues. It is possible that labile interfibrillar cohesion is a phylogenetically ancestral trait, since intact collagen fibrils can be extracted by mild, non-denaturing methods from the collagenous tissues of sponges [101], cnidarians [102] and molluscs [103]. If this is the case, then (1) permanently stable interfibrillar cohesion represents a derived condition, which may have evolved as a result of the greater forces that collagenous structures had to resist and transmit in vertebrate skeleto-muscular systems, and (2) the evolutionary sequence is repeated during the ontogeny of vertebrate collagenous tissues: during embryonic development, collagen fibrils can initially be extracted from chick tendons by gentle homogenisation, but after 15 days post-fertilisation the tendons become virtually unextractable [98]; this is paralleled by an increase in the mechanical strength from 2 MPa at 14 days post-fertilisation to 58 MPa two days post-hatching [104] (a quantitatively similar change, it should be noted, to that undergone (reversibly) by echinoderm MCTs in a time-scale of seconds!).

It is astonishing that neither the factors responsible for the ontogenetic changes in the interfibrillar cohesion of embryonic vertebrate tendons nor the mechanism of interfibrillar force transfer in adult vertebrate tendons are well understood [75], [105], [106]. Investigation of echinoderm mutable collagenous structures may help to rectify this deficiency: their baseline mechanical state and their maximally stiffened state are at least analogous, and may be homologous, to the fetal and adult states respectively of vertebrate collagenous tissues. The CD of the camarodont echinoid lantern is a particularly appropriate model, in that it is easily accessible, it is amenable to mechanical testing, and it has a relatively simple parallel-fibred organisation comparable to that of mammalian tendon. In this paper we have provided detailed information on the baseline static mechanical properties of the CD, which complements our accounts of its biochemistry, molecular organisation and ultrastructure [7–9], [25], [30]. What is needed next is a thorough genomics- and proteomics-based characterisation of the molecular interactions underpinning its different mechanical states. Our work is intended to provide a solid foundation for such investigations.

## Supporting Information

S1 Dataset Creep. (XLSX)

S2 Dataset Force-extension. (XLSX)

S3 Dataset Ultimate parameters. (XLSX)

S4 Dataset Stress relaxation. (XLSX)

S5 Dataset Hyaluronidase. (XLSX)

S6 Dataset Chondroitinase—creep. (XLSX)

S7 Dataset Chondroitinase—force extension. (XLSX)

S8 Dataset Pharmacological agents. (XLSX)

S9 Dataset Cholinergic response times. (XLSX)

S10 Dataset Tensilin. (XLSX)

S1 Table Creep behaviour of *Paracentrotus lividus* CD ligament and other echinoderm and non-echinoderm collagenous structures. (DOCX)

S2 Table Elastic modulus and ultimate properties of *Paracentrotus lividus* CD ligament and other echinoderm and non-echinoderm collagenous structures. (DOCX)

S3 Table Stress relaxation of mammalian tendons. (DOCX)
