# Supplementary material for: Mechanical Properties of the Compass Depressors of the Sea-Urchin Paracentrotus lividus (Echinodermata, Echinoidea) and the Effects of Enzymes, Neurotransmitters and Synthetic Tensilin-Like Protein
Source: PLoS One. 2015 Mar 18;10(3):e0120339. doi: 10.1371/journal.pone.0120339 (PMC4365025; doi:10.1371/journal.pone.0120339)
Supplement: S1 Table — (DOCX) [file pone.0120339.s011.docx]

**Table S1. Creep behaviour of *Paracentrotus lividus* CD ligament and other echinoderm and non-echinoderm collagenous structures.** Values shown are means and/or ranges. Double hyphens indicate lack of information. Since wallaby tail tendon shows no secondary creep phase, we calculated its coefficient of viscosity, using data provided by Wang & Ker (1995), as stress/minimum strain rate, the latter occurring at the

inflection between the primary and tertiary phases.

| Class and species | Anatomical structure | Stress  (kPa) | Coefficient of  viscosity (MPa.s) | Breakage  strain | Reference |
| --- | --- | --- | --- | --- | --- |
| Asteroidea |  |  |  |  |  |
| *Asterias rubens* | aboral dermis at arm autotomy plane | -- | 0.48 | -- | Wilkie et al., 1990 |
| Echinoidea |  |  |  |  |  |
| *Diadema setosum* | central spine ligament | ca. 100 | 20-5860 | 0.8-3.7 | Motokawa,1983 |
| *Paracentrotus lividus* | compass depressor ligament | 168-1163 | 561; 104-1477 | 3.12; 1.41-4.54 | This paper |
| Holothuroidea |  |  |  |  |  |
| *Stichopus chloronotus* | dermis | 0.06 | 0.059; 0.12-0.32 | ca. 10 | Motokawa, 1982 |
| *Stichopus japonicus* | dermis | 300-3000 | 3.0; 0.076-35 | -- | Motokawa, 1984 |
| *Thyone inermis* | dermis | -- | 5100 | -- | Eylers, 1982 |
| *Eupentacta quinquesemita* | introvert | 30 | 100 | ≤ 9 | Byrne, 1985 |
| Ophiuroidea |  |  |  |  |  |
| *Ophiocomina nigra* | intervertebral ligament | 200-1100 | 2260; 540-7100 | 1.45; 0.64-2.82 | Wilkie, 1984 |
| Mammalia |  |  |  |  |  |
| Rat | skin | 196.2 | 11770 | ca. 0.25 | Tregear, 1966 |
| Rat | newborn skin | 29.43 | 4649 | -- | Harkness & Harkness, 1959 |
| Rat | uterine cervix (pregnant) | 29.43 | 128.9 | -- | Harkness & Harkness, 1959 |
| Wallaby | tail tendon | 3-8×10^4^ | 0.8-30×10^6^ | [0.13^1^] | Wang &Ker, 1995 |

**References:**

Byrne M (1985) The mechanical properties of the autotomy tissues of the holothurian *Eupentacta quinquesemita* and the effects of certain physic-chemical agents. J Exp Biol 117: 69-86.

Eylers JP (1982) Ion-dependent viscosity of holothurian body wall and its implications for the functional morphology of echinoderms. J Exp Biol 99: 1-8.

Harkness MLR, Harkness RD (1959) Effect of enzymes on mechanical properties of tissues. Nature 183: 1821-1822.

Motokawa T (1982) Factors regulating the mechanical properties of holothurian dermis. J Exp Biol 99: 29-41.

Motokawa T (1983) Mechanical properties and structure of the spine-joint central ligament of the sea urchin, *Diadema setosum* (Echinodermata, Echinoidea). J. Zool Lond 201: 223-235.

Motokawa T (1984) The viscosity change of the body-wall dermis of the sea cucumber *Stichopus japonicus* caused by mechanical and chemical stimulation.Comp Biochem Physiol 77A: 419-423.

Tregear RT (1966) Physical Functions of Skin. London: Academic Press. 185pp.

Wang XT, Ker RF (1995) Creep rupture of wallaby tail tendons. J Exp Biol 198: 831-845. [^1^Value taken from one creep curve illustrated in Fig. 3]

Wilkie IC (1984) Variable tensility in echinoderm collagenous tissues: a review. Mar Behav Physiol 11: 1-34.

Wilkie IC, Griffiths GVR, Glennie SF (1990) Morphological and physiological aspects of the autotomy plane in the aboral integument of *Asterias* *rubens* L. (Echinodermata). In: De Ridder C, Dubois P, Lahaye M, Jangoux M, editors. Echinoderm Research. Rotterdam: Balkema. pp. 301-313.
