# Supplementary material for: Mechanical Properties of the Compass Depressors of the Sea-Urchin Paracentrotus lividus (Echinodermata, Echinoidea) and the Effects of Enzymes, Neurotransmitters and Synthetic Tensilin-Like Protein
Source: PLoS One. 2015 Mar 18;10(3):e0120339. doi: 10.1371/journal.pone.0120339 (PMC4365025; doi:10.1371/journal.pone.0120339)
Supplement: S2 Table — (DOCX) [file pone.0120339.s012.docx]

**Table S2. Elastic modulus and ultimate properties of *P. lividus* CD ligament and other echinoderm and non-echinoderm collagenous structures.** Values shown are means and/or ranges. Double hyphens indicate lack of information. Since these mechanical parameters tend to be strain rate-dependent, the experimental strain rates are included, where known.

| Class and species | Anatomical structure | Strain rate  (s^-1^) | Young’s  modulus (MPa) | Tensile  strength (MPa) | Breaking  strain | Reference |
| --- | --- | --- | --- | --- | --- | --- |
| Asteroidea |  |  |  |  |  |  |
| *Echinaster spinulosus* | aboral body wall | 0.01-0.02 | 249-353 | 37-45 | 0.12-0.15 | O’Neill, 1989 |
| *Coscinasterias calamaria* | aboral body wall | 0.09-0.23 | 10.56 | 3.27 | 0.43 | O’Neill & Withers, 1995 |
| *Linckia laevigata* | aboral body wall | 0.0004-0.004 | 20.9-36.0 | 3.65-6.27 | 0.23-0.30 | Motokawa, 2011 |
| Echinoidea |  |  |  |  |  |  |
| *Paracentrotus lividus* | compass depressor ligament | 0.003-0.250 | 18.6; 3.3-44.2 | 8.1; 1.5-23.2 | 1.51; 0.56-6.50 | This paper |
| *Eucidaris tribuloides* | spine ligament | 0.006 | 25 | 8 | -- | Szulgit & Shadwick, 1984 |
| *Eucidaris tribuloides* | spine ligament (in catch state) | -- | 200 | -- | -- | Trotter & Koob, 1989 |
| *Anthocidaris crassispina* | spine ligament (ACh-treated) | 0.005-0.5 | 230-420 | 18-38 | -- | Hidaka & Takahashi, 1983 |
| Holothuroidea |  |  |  |  |  |  |
| *Stichopus chloronotus* | dermis | -- | 0.024 |  |  | Motokawa, 1982 |
| *Holothuria leucospilota* | dermis | -- | 0.42 | -- | -- | Motokawa, 1984 |
| *Actinopyga echinites* | dermis | -- | 1.67 | -- | -- | Motokawa, 1984 |
| *Cucumaria frondosa* | collagen fibril | 0.002-0.006 | 470; 110-1470 | 230; 40-490 | 0.80; 0.33-1.83 | Shen et al., 2010 |
| *Cucumaria frondosa* | collagen fibril | -- | 400-1200 | 500 | -- | Eppell et al., 2006 |
| Ophiuroidea |  |  |  |  |  |  |
| *Ophiocomina nigra* | intervertebral ligament | -- | -- | 6.17; 2.20-11.47 | -- | Wilkie, 1988 |
| Myxini |  |  |  |  |  |  |
| *Myxine glutinosa* | tongue retractor tendon | 0.13-0.25 | 290 | 47.8 | 22 | Summers & Koob, 2002 |
| Mammalia |  |  |  |  |  |  |
| Rat | tail tendon | 0.0013 | 1304 | 40-80 | 0.05-0.17 | Kastelic & Baer, 1980 |
| Rat | collagen fibril | -- | -- | 100-500 | -- | Craig et al., 1989 |
| Cow | digital extensor tendon | 0.1 | 639 | 95.7 | 0.231 | Legerlotz et al., 2013 |
| *Homo sapiens* | patellar tendon | 0.006 | 2,000 | -- | -- | Svensson et al., 2012 |
| *Homo sapiens* | collagen fibril | 0.05 | 2800; 70-5100 | -- | -- | Svensson et al., 2012 |

**References:**

Craig AS, Birtles MJ, Conway JF, Parry DAD (1989) An estimate of the mean length of collagen fibrils in rat tail-tendon as a function of age. Connect Tiss Res 19: 51-62.

Eppell SJB, Smith BN, Kahn H, Ballarini R (2006) Nano measurements with micro-devices: mechanical properties of hydrated collagen fibrils. J R Soc Interface 3: 117–121.

Hidaka M, Takahashi K (1983) Fine structure and mechanical properties of the catch apparatus of the se-urchin spine, a collagenous connective tissue with muscle-like holding capacity. J Exp Biol 103: 1-14.

Kastelic J, Baer E (1980) Deformation in tendon collagen. In: Vincent JFV, Currey JD, editors. Mechanical Properties of Biological Materials. Cambridge: Cambridge University Press. pp. 397-435.

Legerlotz K, Riley GP, Screen HRC (2013) GAG depletion increases the stress-relaxation response of tendon fascicles, but does not influence recovery. Acta Biomaterialia 9: 6860-6866.

Motokawa T (1982) Factors regulating the mechanical properties of holothurian dermis. J Exp Biol 99: 29-41.

Motokawa T (2011) Mechanical mutability in connective tissue of starfish body wall. Biol Bull 221: 280-291.

O’Neill PL (1989) Structure and mechanics of starfish body wall. J Exp Biol 147: 53-89.

O’Neill PL, Withers PC (1995) An analysis of the load curve of the body wall of *Coscinasterias calamaria* (Echinodermata: Asteroidea). Mar Fresh Behav Physiol 25: 245-260.

Shen ZL, Dodge MR, Kahn H, Ballarini R, Eppell SJ (2010) In vitro fracture testing of submicron diameter collagen fibril specimens. Biophys J 99: 1986-1995.

Summers AP, Koob TJ (2002) The evolution of tendon – morphology and material properties. Comp Biochem Physiol 133A:1159-1170.

Svensson RB, Hansen P, Hassenkam T, Haraldsson BT, Aagaard P, Kovanen V, Krogsgaard M, Kjaer M, Magnusson SP (2012) Mechanical properties of human patellar tendon at the hierarchical levels of tendon and fibril. J Appl Physiol 112: 419-426.

Szulgit GK, Shadwick RE (1994) The effects of calcium chelation and cell perforation on the mechanical properties of sea urchin ligaments. In: David B, Guille A, Féral JP, Roux M, editors. Echinoderm through Time. Rotterdam: Balkema. pp. 887-892.

Trotter JA, Koob TJ (1989) Collagen and proteoglycan in a sea urchin ligament with mutable mechanical properties. Cell Tiss Res 258: 527-539.

Wilkie IC (1988) Design for disaster: the ophiuroid intervertebral ligament as a typical mutable collagenous structure. In: Burke RD, Mladenov PV, Lambert P, Parsley RL, editors. Echinoderm Biology. Rotterdam: Balkema. pp. 25-38.
