# Supplementary material for: Mechanical Properties of the Compass Depressors of the Sea-Urchin Paracentrotus lividus (Echinodermata, Echinoidea) and the Effects of Enzymes, Neurotransmitters and Synthetic Tensilin-Like Protein
Source: PLoS One. 2015 Mar 18;10(3):e0120339. doi: 10.1371/journal.pone.0120339 (PMC4365025; doi:10.1371/journal.pone.0120339)
Supplement: S3 Table — (DOCX) [file pone.0120339.s013.docx]

| Tendon | Stress relaxation_0-10s_  (% peak stress) | Constant  strain | Comments | Reference |
| --- | --- | --- | --- | --- |
| Rat tail | 9 | 0.035 |  | Rigby et al., 1959: Fig. 7 |
| Rat tail | 17 | 0.075 |  | Rigby et al., 1959: Fig. 7 |
| Human patellar | 20 | 0.02 | small specimens | Atkinson et al., 1999: Fig. 6 |
| Human Patellar | 50 | 0.02 | large specimens | Atkinson et al., 1999: Fig. 6 |
| Porcine digital flexor | 33 | 0.06 |  | Duenwald et al., 2009: Fig. 4 |
| Porcine digital flexor | 25 | 0.05 |  | Duenwald et al., 2009: Fig. 4 |

**Table S3. Stress relaxation of mammalian tendons.** Stress relaxation occurring 0-10 s after the peak stress, expressed as a percentage of the peak stress.

**References:**

Atkinson TS, Ewers BJ, Haut RC (1999) The tensile and stress relaxation responses of human patellar tendon varies with specimen cross-sectional area. J Biomech 32: 907-914.

Duenwald SE, Vanderby R, Lakes RS (2009) Viscoelastic relaxation and recovery of tendon. Ann Biomed Eng 37: 1131-1140.

Rigby BJ, Hirai N, Spikes JD, Eyring H (1959) The mechanical properties of rat tail tendon. J Gen Physiol 43: 265-283.
